# Application of a Transfer Learning Model Combining CNN and Self-Attention Mechanism in Wireless Signal Recognition

**DOI:** 10.3390/s25134202

**Published:** 2025-07-05

**Authors:** Wu Wei, Chenqi Zhu, Lifan Hu, Pengfei Liu

**Affiliations:** 1 Nanjing Cowave Communication Technology Co., Ltd., Nanjing 211135, China; wave@cowave.com; 2 College of Communication and Information Engineering, Nanjing University of Posts and Telecommunications, Nanjing 210003, China; 1022010103@njupt.edu.cn (C.Z.); pfliu@njupt.edu.cn (P.L.)

**Keywords:** convolutional neural network, self-attention, wireless signal recognition, low SNR, low sampling rate

## Abstract

In this paper, we propose TransConvNet, a hybrid model combining Convolutional Neural Networks (CNNs), self-attention mechanisms, and transfer learning for wireless signal recognition under challenging conditions. The model effectively addresses challenges such as low signal-to-noise ratio (SNR), low sampling rates, and limited labeled data. The CNN module extracts local features and suppresses noise, while the self-attention mechanism within the Transformer encoder captures long-range dependencies in the signal. To enhance performance with limited data, we incorporate transfer learning by leveraging pre-trained models, ensuring faster convergence and improved generalization. Extensive experiments were conducted on a six-class wireless signal dataset, downsampled to 1 MSPS to simulate real-world constraints. The proposed TransConvNet achieved 92.1% accuracy, outperforming baseline models such as LSTM, CNN, and RNN across multiple evaluation metrics, including RMSE and $R^{2}$. The model demonstrated strong robustness under varying SNR conditions and exhibited superior discriminative ability, as confirmed by Precision–Recall and ROC curves. These results validate the effectiveness and robustness of the TransConvNet model for wireless signal recognition, particularly in resource-constrained and noisy environments.

## 1. Introduction

Wireless signal recognition has become a critical component in a wide range of applications, including 5G communication systems, the Internet of Things (IoT), and vehicular networks [1]. Accurate recognition of wireless signals allows for efficient spectrum management, interference mitigation, and enhanced communication reliability [2]. With the rapid development of modern wireless communication technologies, the volume of data and the complexity of signal environments have increased, presenting new challenges for wireless signal recognition systems.

In particular, the task of recognizing wireless signals in real-world scenarios is often complicated by issues such as low sampling rates, low signal-to-noise ratio (SNR), and limited availability of labeled data. Traditional methods based on handcrafted features and shallow learning models are often insufficient to handle these challenges [3,4,5,6]. In contrast, deep learning models, which automatically learn relevant features from raw data, have shown great promise in improving the accuracy and robustness of wireless signal recognition [7,8,9,10,11]. To address the limitations of individual architectures, numerous hybrid structures combining the strengths of different deep learning approaches have been proposed under comparable constraints [12,13]. These hybrid methods typically integrate convolutional neural networks for local feature extraction with attention mechanisms or recurrent structures for capturing long-range dependencies, demonstrating improved performance in challenging signal environments. However, deep learning models typically require large amounts of labeled data to achieve optimal performance, which is often not feasible in practical situations [14]. In real applications, the available labeled data is usually limited, making it difficult to train deep models from scratch [15]. Transfer learning has emerged as an effective approach to alleviate this problem [16,17]. Transfer learning leverages pre-trained models from related tasks to achieve faster convergence and better generalization. This approach is especially useful in wireless signal recognition, where obtaining large amounts of labeled data is often expensive and time-consuming [18,19,20,21].

In this work, we propose a new approach combining CNN, self-attention mechanisms, and transfer learning to optimize wireless signal recognition, especially in low sampling rate and low SNR conditions. The core of our model architecture consists of a convolutional transformer network (TransConvNet), where CNN layers are used for initial feature extraction and noise suppression, followed by a transformer encoder with a self-attention mechanisms to capture long-range dependencies in the signal. In addition, we apply transfer learning to improve the generalization ability of the model, ensuring robust performance even when labeled data is scarce. The main contributions of this paper are as follows:

- Proposed TransConvNet model: We design a hybrid architecture that combines CNN and self-attention mechanism to achieve effective signal recognition, capturing local and global dependencies in signal data.
- Optimization for low signal-to-noise ratio and low sampling rate: We pay special attention to optimizing signal recognition under low sampling rates and noisy environments, where traditional methods often perform poorly.
- Transfer learning: We incorporate transfer learning to leverage pre-trained models, allowing our method to achieve superior performance even with limited training data.
- Comprehensive evaluation: We evaluate our model on a real-world wireless signal dataset and demonstrate that it significantly outperforms baseline models in terms of accuracy, robustness, and noise resistance.

The rest of this paper is organized as follows. Section 2 reviews the related work on wireless signal recognition, CNN, self-attention mechanism, and transfer learning. Section 3 describes the detailed architecture and methodology of the proposed model. In Section 4, we present the experimental setup, results, and discussion. Finally, Section 5 concludes this paper and outlines future research directions.

## 2. Related Work

### 2.1. Wireless Signal Recognition

Wireless signal recognition plays a crucial role in modern communication systems. Traditional methods often rely on manual feature engineering and classical machine learning algorithms such as Support Vector Machines (SVM) and k-Nearest Neighbors (k-NN) [22]. Nandi et al. [23] employed a decision-theoretic approach to develop a set of decision criteria for the identification of different modulation types. Experimental results demonstrated that when the SNR was 15 dB, the overall accuracy exceeded 94%. Wang et al. [24] proposed a novel digital modulation recognition algorithm based on higher-order cumulants (HOC) and support vector machines (SVM), utilizing a binary tree SVM as the classifier. Experimental results indicated that when the SNR was 10 dB, the recognition accuracy surpassed 97.5%. While effective in certain scenarios, these methods struggle in noisy environments and with high-dimensional data. Recently, deep learning approaches, particularly CNNs and Recurrent Neural Networks (RNNs), have demonstrated superior performance by automatically extracting features [25]. Yashashwi et al. [26] proposed a signal distortion correction module (CM) designed for end-to-end joint training with CNN. By utilizing the output of the CM to adjust the signal’s frequency and phase prior to modulation recognition, effective correction can be achieved. Li et al. [27] introduced a novel very high frequency (VHF) radio signal modulation recognition algorithm based on antinoise processing and deep sparse-filtering convolutional neural network (AN-SF-CNN). Simulation results demonstrated that the proposed method achieves higher or comparable classification accuracy and exhibits robustness against noise when compared with traditional approaches.

### 2.2. Convolutional Neural Networks in Signal Processing

CNNs have been widely adopted in signal processing due to their ability to capture local patterns through convolution operations. They effectively handle time- and frequency features of signals, making them suitable for noise suppression and feature extraction [28]. Kulin et al. [29] evaluated the performance of three CNN structures using time–IQ data, amplitude/phase representations, and frequency representations. Experimental results showed that, compared with IQ and frequency data, employing amplitude/phase representations for modulation format recognition improved performance by 2% and 12%, respectively, at medium and high SNR. Selim et al. [30] proposed a data preprocessing method that utilizes the amplitude and phase shift of collected samples. This approach enabled the CNN to achieve a classification accuracy of 99.6%. However, conventional CNNs are limited in capturing long-range dependencies and global context, which are critical in recognizing complex signal patterns.

#### 2.2.1. Self-Attention Mechanisms and Transformers

Self-attention mechanisms dynamically assign weights to input features, allowing models to capture global dependencies. Originally introduced in natural language processing tasks, Transformer models have shown remarkable success in handling sequential data. In the context of wireless signal recognition, self-attention can effectively model the temporal relationships across signal sequences. Liang et al. [31] proposed a DL-based end-to-end automatic modulation recognition (AMR) framework named CCNN-Atten. This approach employs a complex-valued convolutional neural network for feature extraction, combined with feature calibration and an improved multi-head attention mechanism, which effectively enhances feature representation and temporal dependency modeling capabilities. Experimental results demonstrate that CCNN-Atten achieves 1–10% higher recognition accuracy and lower computational complexity than current state-of-the-art (SOTA) methods across multiple public datasets. Wei et al. [32] proposed an end-to-end sequence-based network that integrates a shallow convolutional neural network, bidirectional long short-term memory (Bi-LSTM), and a self-attention mechanism for recognizing eight kinds of intrapulse modulations of radar signals. Experimental results show that the proposed network achieves over 95% at −10 dB. Furthermore, it outperforms four existing methods in terms of recognition performance and computational complexity, especially in low SNR scenarios. However, its high computational cost makes direct application to low-dimensional signal data challenging, necessitating hybrid architectures combining attention mechanisms with other efficient feature extraction techniques.

#### 2.2.2. Transfer Learning in Wireless Signal Recognition

Transfer learning has emerged as a powerful approach for tasks with limited labeled data, enabling faster convergence and improved generalization by leveraging pre-trained models [33]. In wireless signal recognition, where acquiring large-scale labeled datasets is expensive and time-consuming, transfer learning offers a practical solution [34]. Xiao et al. [35] proposed an automatic radar waveform recognition system based on Choi–Williams distribution (CWD) time-frequency images and deep learning, transforming the signal recognition problem into an image recognition task. By adopting transfer learning and integrating both texture features and deep features, the method effectively improves recognition performance with small-scale datasets. Experimental results demonstrate that this approach can achieve effective radar signal recognition under low SNR conditions. An et al. [36] proposed a transfer learning framework for human activity recognition (HAR). By analyzing transferable and user-specific features, the framework enables partial migration and fine-tuning of the classifier. Experimental results show that, compared with the baseline without transfer learning, the proposed method improves recognition accuracy by up to 43%, reduces training time by 66%, and lowers power and energy consumption by 43% and 68%, respectively. Most existing work focuses on using pre-trained models for image or language tasks, with limited exploration in signal processing, especially under low SNR and low sampling rate scenarios. Further research is required to adapt transfer learning to these unique challenges [37,38,39].

## 3. Methodology

### 3.1. Data Preprocessing

A comprehensive data preprocessing pipeline is designed for the collected original samples to improve classification accuracy under different signal-to-noise ratio conditions. First, to simulate various channel conditions in real-world environments, Gaussian white noise is added to the original signals, covering an SNR range from −15 dB to 25 dB. The mathematical expression is as follows:

(1) $$R\left[n\right]=R\left[n\right]+W_{SNR,n},\;n=1,2,\ldots ,N$$

where R[n] represents the original signal, $W_{SNR,n}$ represents the Gaussian white noise generated according to the target SNR, and *N* is the number of samples at the set sampling rate.

Subsequently, the in-phase and quadrature components are extracted from each complex sample, namely the real and imaginary parts, to restore the time representation of the signal. The specific calculation formula is

(2) I[n]=Re(R[n]),Q[n]=Im(R[n]),n=1,2,…,N

where I[n] and Q[n] represent the in-phase and quadrature components of the *n*-th sample, respectively. Considering that the characteristics of time–IQ signals may be masked by noise under low SNR conditions, this paper further transforms the signal to the frequency domain to enhance feature discriminability. Fast Fourier Transform is applied to the IQ samples within each Time Resource Window for frequency domain conversion. The transformation formula is as follows:

(3) $$R^{\prime \prime }\left[k\right]=\sum_{i=1}^{M}\left(I\left[i\right]+jQ\left[i\right]\right)e^{-j\frac{2\pi (i-1)(k-1)}{M}},\;k=1,2,\ldots ,M$$

where *M* is the number of sample points included in the TRW under the current sampling rate. For example, at a 5 Msps sampling rate, a 44 µs TRW corresponds to M=220. $R^{\prime \prime }\left[k\right]$ represents the *k*-th frequency domain point. After the Fast Fourier Transform, the complex results are separated into real and imaginary parts and arranged to form the input matrix for the neural network model:

(4) It=Re(R″[1])Re(R″[2])⋯Re(R″[M])Im(R″[1])Im(R″[2])⋯Im(R″[M])

This frequency feature representation not only has higher discriminability under low SNR conditions, but also provides stable and distinguishable input data for subsequent convolutional feature extraction modules. After completing the above preprocessing, signal samples are used for model training and inference stages to achieve recognition and communication behavior feature extraction for multiple wireless technologies.

### 3.2. Model Architecture

**Figure 1:**
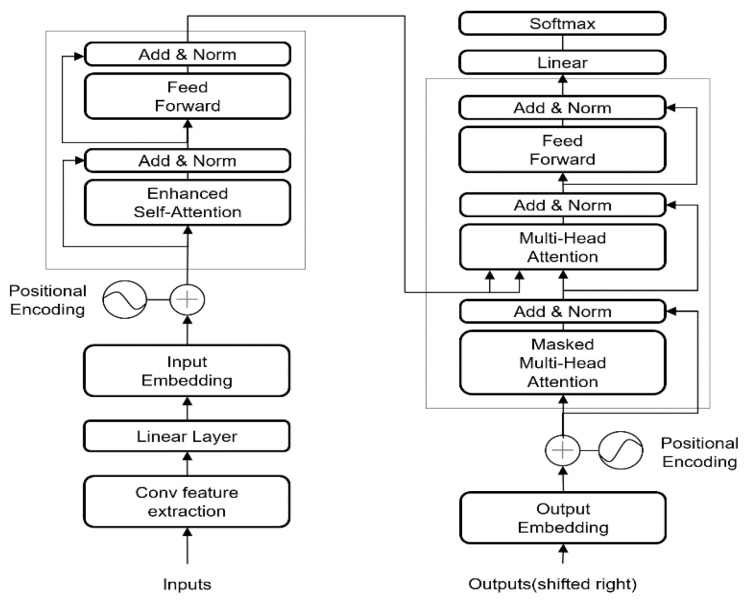
The proposed TransConvNet architecture.

The proposed TransConvNet comprises three core components: CNN module, self-attention mechanism, and transfer learning strategy, which work synergistically to address the challenge of constraints in wireless signal recognition. Figure 1 illustrates the structure of the model.

The CNN module is responsible for extracting robust, noise-resistant local features and providing hierarchical feature abstraction. This module employs multi-scale convolutional filters to capture local signal patterns, effectively handling both time- and frequency features, while max-pooling operations reduce noise sensitivity and provide a stable, reliable feature foundation for subsequent global dependency modeling. The self-attention mechanism employs multi-head attention computation. This mechanism computes weighted relationships between all time points, enabling the model to dynamically focus on relevant distant features, effectively overcoming the limited receptive field problem of traditional CNNs and achieving direct modeling of arbitrary–distance feature relationships. The transfer learning strategy enhances model performance through pre-trained weight initialization. Specifically, CNN layers are initialized with pre-trained weights from related signal processing tasks to ensure robust local feature extraction, while Transformer layers utilize pre-trained weights from general-purpose or domain-specific models. Through this synergistic mechanism, the model achieves effective feature extraction and dependency modeling even with limited labeled data, significantly improving convergence speed and generalization performance.

#### 3.2.1. CNN Feature Extraction

**Figure 2:**
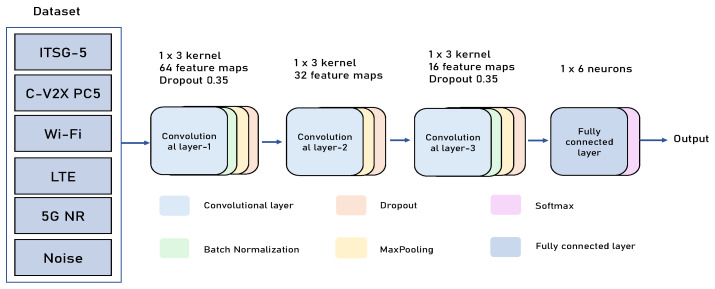
The proposed CNN architecture.

The CNN module extracts local features and suppresses noise in the input signal. As shown in Figure 2, the CNN module consists of three convolutional layers with the following configurations: the first convolutional layer employs 64 filters with a kernel size of 1 × 3, stride of 1, and padding of 2, resulting in feature dimensions of 64 × (M + 2); the second convolutional layer uses 32 filters with a kernel size of 1 × 3 and padding of 2, producing feature dimensions of 32 × (M + 4); the third convolutional layer applies 16 filters with a kernel size of 1 × 3 to extract higher-order features.

The operation form of each convolutional layer is

(5) $$X^{\left(l\right)}=\sigma \left(W^{\left(l\right)}*X^{\left(l-1\right)}+b^{\left(l\right)}\right)$$

where $X^{\left(l\right)}$ represents the output feature map of the *l*-th layer, $W^{\left(l\right)}$ is the convolutional kernel weights, * denotes the one-dimensional convolution operation, and σ is the activation function.

Rectified Linear Unit (ReLU) is used to enhance non-linear expression capability, where $b^{\left(l\right)}$ is the bias term. Each convolutional layer is followed by Batch Normalization (BN) operations to accelerate convergence and improve stability. Additionally, a max-pooling layer is set after each convolutional layer for downsampling features, reducing computational complexity, and enhancing translation invariance. The feature maps are input to the first fully connected layer containing 1024 neurons after flattening operations, using the ReLU activation function.

The module extracts signal local features, partial distribution, and edge-changing short-term structural information, providing high-quality representations for subsequent overall modeling.

#### 3.2.2. Transformer Encoding:

The feature map output by the CNN is reshaped and passed through a Transformer encoder to capture long-range dependencies.

**Reshape Operation:**

(6) $$Z=\mathrm{Reshape}\left(y_{2,\mathrm{pooled}}\right)\to R^{N\times S\times D}$$

where *S* is the sequence length after CNN processing and *D* is the feature dimension.

**Multi-Head Self-Attention**

(7) $$\mathrm{Attention}\left(Q,K,V\right)=\mathrm{softmax}\left(\frac{QK^{T}}{\sqrt{d_{k}}}\right)V$$

(8) $$\mathrm{MultiHead}\left(Z\right)=\mathrm{Concat}\left({\mathrm{head}}_{1},{\mathrm{head}}_{2},\ldots ,{\mathrm{head}}_{h}\right)W^{O}$$

where ${\mathrm{head}}_{i}=\mathrm{Attention}\left(ZW_{i}^{Q},ZW_{i}^{K},ZW_{i}^{V}\right)$.

**Feed-Forward Network**

(9) $$\mathrm{FFN}\left(x\right)=\max\left(0,xW_{1}+b_{1}\right)W_{2}+b_{2}$$

**Transformer Layer Output:**

(10) Output=LayerNorm(Z+MultiHead(Z))

(11) Output=LayerNorm(Output+FFN(Output))

#### 3.2.3. Classification Layer

**Global Average Pooling:**

(12) $$z_{\mathrm{pooled}}=\frac{1}{S}\sum_{i=1}^{S}{\mathrm{Output}}_{i}$$

**Final Classification:**

(13) $$\hat{y}=\mathrm{softmax}\left(W_{c}\cdot z_{\mathrm{pooled}}+b_{c}\right)$$

where $W_{c}\in R^{D\times C}$ and $b_{c}\in R^{C}$ are the classification layer parameters, and *C* is the number of signal classes.

### 3.3. Transfer Learning Strategy

The transfer learning strategy employed in TransConvNet leverages pre-trained weights to enhance performance and accelerate convergence. Specifically, the CNN module is initialized with weights from a pre-trained model on a related dataset to ensure robust local feature extraction, while the Transformer encoder utilizes pre-trained weights from a general-purpose model such as BERT or a domain-specific signal processing model. During the fine-tuning process, certain layers are selectively frozen or updated to optimize task-specific performance. The early CNN layers are frozen to preserve general feature extraction capabilities, while the Transformer layers are partially updated to focus on capturing domain-specific long-range dependencies. This approach ensures efficient transfer of knowledge while adapting to the unique characteristics of the target task.

Mathematically, given a loss function L(θ), the model’s parameters are updated as:

(14) $$\theta_{t+1}=\theta_{t}-\eta \nabla_{\;\theta }\;L\left(\theta \right)$$

where η is the learning rate. Frozen layers have $\nabla_{\;\theta }\;L\left(\theta \right)=0$.

## 4. Experiments

### 4.1. Experimental Setup

#### 4.1.1. Dataset and Preprocessing

The dataset utilized in this study comprises six distinct categories: CV2X, 5G, ITSG5, LTE, Noise, and Wi-Fi, representing various wireless signal types and noise commonly encountered in real-world communication systems.

In particular, the collection of CV2X and ITS-G5 signals employs the CAMINO framework, which provides robust support for managing multiple V2X communication technologies. The acquisition process operates at a smart highway testbed near Antwerp, Belgium, equipped with eight roadside units (RSUs) and two onboard units (OBUs). Standardized C-ITS service messages are dynamically generated via the CAMINO framework (University of Antwerp, Antwerp, Belgium), enabling the collection of diverse V2X communication signal samples under real-world road traffic conditions.

For 5G signal generation, the Open Air Interface platform (Eurecom, Sophia Antipolis, France) operates in non-standalone mode, leveraging existing LTE infrastructure to support 5G communication. The system configuration includes Time Division Duplex (TDD) static configuration 1, characterized by a slot ratio of 1:1, a center frequency of 5.9 GHz, a bandwidth of 10 MHz, and subcarrier spacing based on Numerology 1. The MCS index range spans from 1 to 28, covering a load range of 5 Mbps to 50 Mbps, thereby obtaining 5G NR signal samples with varying coding rates and modulation schemes.

LTE signal generation utilizes the open-source software-defined radio platform srsRAN (https://www.srslte.com, accessed on 17 May 2022). A complete LTE link is established, and data collection is performed in an indoor test environment. The acquisition system relies on USRP X310 devices (Ettus Research, Santa Clara, CA, USA), configured with a center frequency of 5.9 GHz, a bandwidth of 10 MHz, and operating in Frequency Division Duplex (FDD) mode. To simulate different network load conditions, the modulation and coding scheme is manually adjusted during the experiment, with indices ranging from 1 to 28 and traffic loads spanning 5 Mbps to 50 Mbps, generating diverse LTE signal samples.

Noise signals are categorized as an independent dataset for training the wireless technology identification model. Given that noise floor levels are significantly influenced by hardware states and device types, traditional threshold-based detection methods are insufficient for accurate differentiation. Therefore, noise samples are collected separately. The collection is performed on clean channels free from other wireless transmission interference, using USRP X310 devices in indoor environments and USRP N310 devices (Ettus Research, Santa Clara, CA, USA) deployed at the smart highway testbed for outdoor environments. To ensure coverage of different sampling rates, noise signal samples are independently collected for each preset sampling rate, comprehensively enhancing the dataset’s generalization capability and the model’s discriminative robustness.

Wi-Fi signal collection is based on the openwifi platform, which implements a complete IEEE 802.11a/g/n protocol stack on the Xilinx Zynq SoC architecture (Xilinx Inc., San Jose, CA, USA). An IEEE 802.11n access point and client are set up, with data traffic controlled at 10 to 200 packets per second and packet sizes varying between 500 and 1500 bytes. By adjusting the MCS index within the range of 0 to 7, Wi-Fi communication data covering different modulation schemes and coding rates are generated, ensuring the diversity and completeness of the signal samples.

Moreover, to ensure that the wireless technology identification model possesses sufficient temporal resolution and practical deployment applicability in mixed time-frequency environments, this study adopts a fixed-length Temporal Resolution Window (TRW) during feature extraction and model identification. The TRW must be no shorter than the minimum frame duration among all candidate communication protocols to avoid feature loss caused by signal truncation. Among the six target signal types, the ACK acknowledgment frame of the IEEE 802.11n protocol is the shortest known frame, with a duration of 44 µs, and is widely present in Wi-Fi environments. Thus, 44 µs can be considered the theoretical minimum granularity for the presence of valid frame structures in the channel, serving as a lower-bound reference for TRW design. In the 5.9 GHz ITS band, where multiple wireless communication technologies coexist, an excessively long TRW (e.g., 100 µs or 244 µs) would significantly increase the probability of multiple protocol frames overlapping within the same window. This could lead to mislabeling of mixed signals and blurred classification boundaries, thereby affecting signal identification and subsequent traffic characterization accuracy. Particularly in safety-critical communication scenarios like V2X, which are highly sensitive to latency and misjudgment, a long TRW may also introduce potential collision risks. Therefore, a short TRW design offers clear advantages in technology identification tasks. Considering identification accuracy, processing complexity, and time-interference characteristics in multi-technology coexistence scenarios, the TRW length is ultimately set to 44 µs.

In addition, to evaluate the model’s generalization capability, the dataset was divided into training and testing subsets with a 70:30 split, ensuring that 30% of the data remained unseen during training. Collected at a high sampling rate of 25 MSPS, the dataset captures detailed signal features representative of typical wireless communication scenarios. Preprocessing steps included normalizing the signal amplitudes to the range [0, 1] to standardize the inputs for the neural network and improve training stability.

After training, the model was evaluated on a testing dataset downsampled to 1 MSPS, simulating low-sampling-rate conditions where signals contain less detail and fewer high-frequency components. This downsampling step tests the model’s ability to generalize and perform well under more practical, resource-constrained scenarios, such as when operating in low-bandwidth or low-complexity environments. Before feeding the data into the model, the **In-phase** component (I[n]) and **Quadrature-phase** component (Q[n]) of components of each sample were extracted. These represent the real and imaginary parts of the sampled signal. While IQ values can be used directly, studies show that Fast Fourier Transform Algorithm (FFT) of IQ values leads to better classification accuracy, especially in low Signal-to-Noise Ratio (SNR) conditions, as FFT captures frequency features that are more distinguishable. Although FFT introduces additional computational complexity, it is essential for high classification accuracy, particularly for Intelligent Transportation Systems (ITS) applications, where safety-critical data is transmitted. Therefore, we computed the FFT of the IQ components, normalized the resulting frequency features to a range of [0, 1], and used these as input for training the neural network. After training, the model was evaluated on a test set downsampled to 1 MSPS to simulate low-sampling-rate conditions.

#### 4.1.2. Data Augmentation and Noise Handling

To enhance the model’s robustness against noise and improve its generalization in real-world environments, several data augmentation techniques were applied during training. Gaussian noise was added to the signals to simulate typical interference found in wireless communication systems. Additionally, signal perturbations, including slight variations in frequency and phase, were introduced to mimic real-world channel distortions.

To further improve signal quality, a DnCNN denoising model [40] was employed to preprocess the training data, removing additive noise and improving signal clarity. This preprocessed, denoised data helps the model focus on the essential features of the signal while reducing the impact of noise during training.

#### 4.1.3. Model Configuration

The model utilized in this study is built on the TransConvNet architecture, which integrates convolutional layers for local feature extraction with the self-attention mechanism of the Transformer for modeling long-range dependencies. The architecture includes two 1D convolutional layers, each followed by ReLU activation and max-pooling operations, which are designed to extract relevant features from the signal while suppressing noise. These features are then passed through a series of Transformer encoder layers equipped with multi-head self-attention mechanisms, enabling the model to capture temporal dependencies across the signal sequence. Finally, a fully connected layer performs the classification, outputting the predicted signal category. To enhance performance and facilitate faster convergence, the model is initialized with pre-trained weights from a related signal processing task, leveraging transfer learning to address the challenges of limited labeled data.

#### 4.1.4. Training Procedure

The TransConvNet model was trained using the Adam optimizer [41] with a learning rate of 0.0001 and a batch size of 16. The training process was run for a maximum of 500 epochs, with early stopping implemented to prevent overfitting. Cross-entropy loss [42] was used as the objective function for multi-class classification.

During training, the model’s weights were updated through backpropagation, with frozen layers in the CNN and Transformer modules to retain general feature extraction knowledge from pre-trained models. Fine-tuning was performed on the remaining layers to adapt the model to the specific wireless signal recognition task. This approach helped the model generalize better, particularly when dealing with the low-sampling-rate data after transfer learning from a higher-sampling-rate environment.

#### 4.1.5. Testing and Evaluation Metrics

After training, the model was evaluated on a test set downsampled to 1 MSPS to assess its ability to generalize to lower-resolution signals. The evaluation included multiple metrics to comprehensively analyze performance: Accuracy measured the percentage of correctly classified signals, while Root Mean Square Error (RMSE) [43] assessed the regression precision of continuous predictions. The Coefficient of Determination ($R^{2}$) [44] evaluated how well the model explained variance in the data, with higher values indicating a better fit. Variance was analyzed to assess the model’s stability, where lower values signified greater robustness. Additionally, the Confusion Matrix was used to examine classification performance across signal categories and identify misclassifications. To evaluate the model’s handling of class imbalances and its discriminative ability, Precision–Recall (PR) curves and Receiver Operating Characteristic (ROC) Curves [45] were employed, providing insights into the trade-off between precision, recall, and false positive rates.

### 4.2. Results and Analysis

The TransConvNet was evaluated on the test set downsampled to 1 MSPS, and it outperformed all baseline models across multiple evaluation metrics. The model achieved an impressive accuracy of 92.1%, which is significantly higher than the performance of the baseline models. Furthermore, the Root Mean Square Error (RMSE) for TransConvNet was 0.84, indicating it made more precise predictions with lower error compared with the other models. In addition, the $R^{2}$ for TransConvNet was 0.45, which is higher than the other models, suggesting a better fitting effect of the proposed method.

The performance comparison for all models tested on the 1 MSPS downsampled dataset is summarized in Table 1.

**Figure 3:**
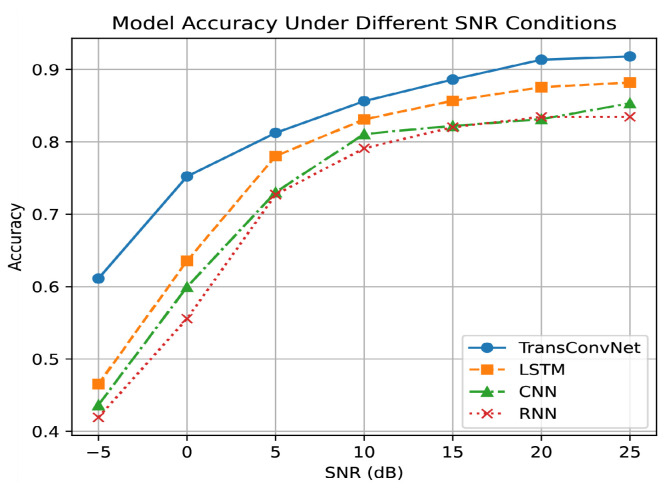
Model accuracy under different SNR conditions.

To evaluate the robustness of the proposed TransConvNet under varying SNR conditions, we compared its performance with baseline models at SNR levels ranging from −5 dB to 25 dB. The accuracy of each model at different SNR values is illustrated in Figure 3.

The TransConvNet model outperforms the baseline models across all SNR levels. Even under extremely low SNR conditions, TransConvNet demonstrates remarkable robustness to noise, maintaining significantly higher accuracy compared with other models.

As the SNR improves, the accuracy of all models increases; however, TransConvNet consistently leads, showing its superior ability to extract both local and global features from the signal. This result highlights the effectiveness of combining CNN-based feature extraction with Transformer-based long-range dependency modeling, enabling the proposed architecture to perform reliably in challenging low-SNR environments.

**Figure 4:**
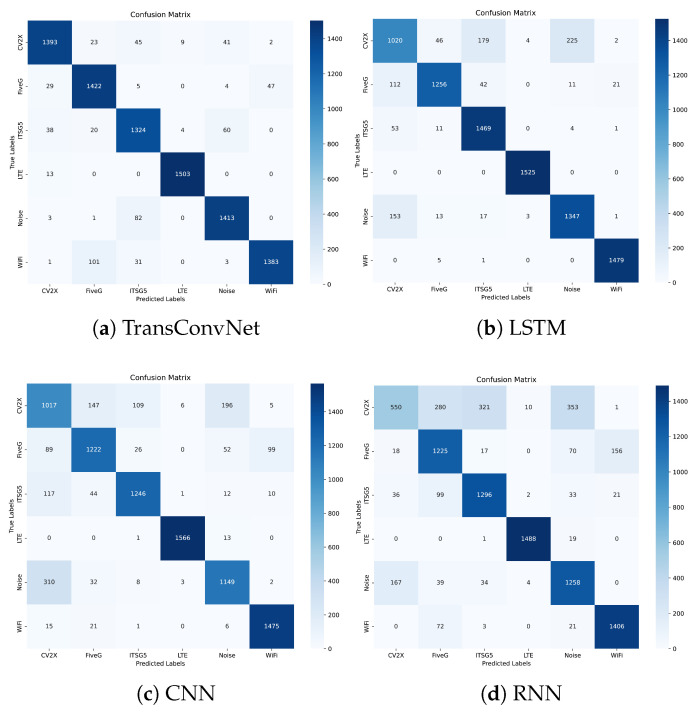
Performance comparison: (**a**) TransConvNet, (**b**) LSTM, (**c**) CNN, and (**d**) RNN.

Confusion matrices are used to demonstrate the performance of each model in recognizing six wireless signal classes [46]. The proposed TransConvNet combines the local feature extraction power of convolutional layers with the global attention mechanism of Transformer encoders. Figure 4 reveals that the TransConvNet model achieves superior classification performance, reducing misclassifications significantly. Notably, CV2X, LTE, and Wi-Fi classes exhibit higher recognition accuracy, with fewer instances of misclassification.

**Figure 5:**
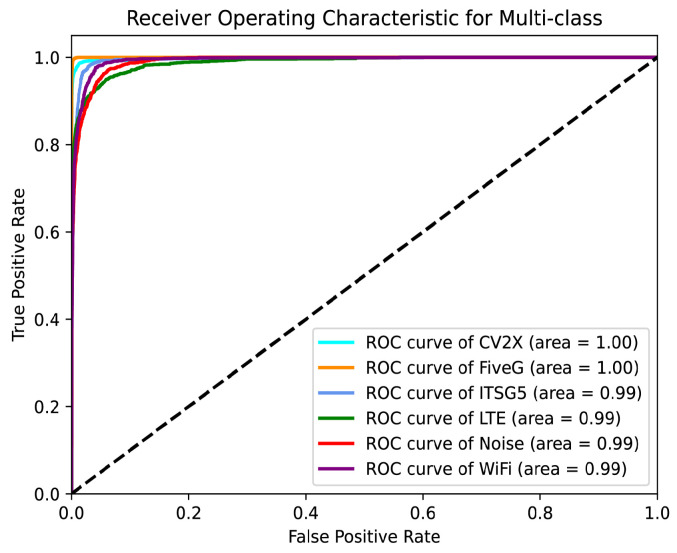
Precision–Recall curves for TransConvNet.

**Figure 6:**
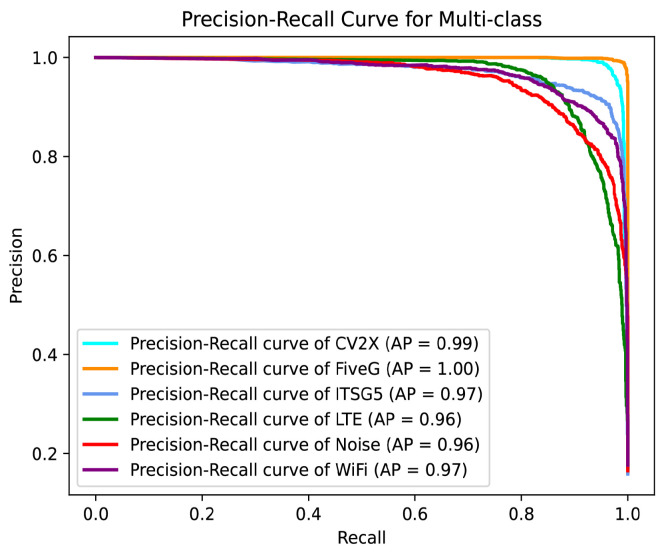
Receiver Operating Characteristic curves for TransConvNet.

Figure 5 and Figure 6 illustrate the performance of the proposed model in the multi-class wireless signal recognition task using the PR curves and ROC curves, respectively.

The PR curves show that the model maintains high precision across all six classes while achieving high recall. These results highlight the model’s ability to accurately identify positive samples and its robustness in maintaining high precision even as recall increases. The ROC curves demonstrate the model’s ability to distinguish between the six signal classes. All ROC curves are close to the top-left corner, and the Area Under the Curve (AUC) values for all classes are exceptionally high. The CV2X and FiveG classes achieve a perfect AUC of 1.00, while the remaining classes achieve AUC values of 0.99. These results indicate that the model has excellent discriminative power and a low false positive rate across all signal classes.

**Figure 7:**
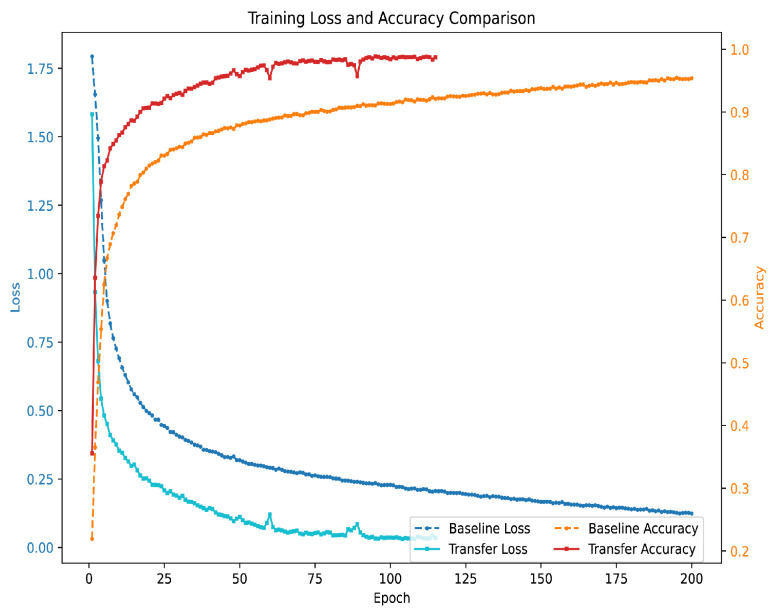
Receiver Operating Characteristic curves for TransConvNet.

The effectiveness of transfer learning is particularly evident when training on small datasets. With only 6 classes and 100 samples per class, the model using transfer learning achieves faster convergence and higher accuracy compared with training from scratch. As shown in Figure 7, transfer learning significantly reduces the training time and loss, allowing the model to reach optimal performance with fewer epochs. This highlights the advantage of leveraging pre-trained weights, especially when labeled data is limited.

The results demonstrate that the proposed TransConvNet model outperforms baseline models across multiple metrics, including accuracy, RMSE, and $R^{2}$. It exhibits strong robustness under varying SNR conditions, maintaining high accuracy even in low-SNR environments. Additionally, the use of transfer learning significantly enhances model performance, especially with limited labeled data, allowing for faster convergence and better generalization. These findings underscore the effectiveness of the TransConvNet architecture in wireless signal recognition tasks.

## 5. Conclusions

In this work, we introduced TransConvNet, a novel hybrid model that integrates CNN-based local feature extraction, Transformer-based global dependency modeling, and transfer learning to optimize wireless signal recognition. The proposed architecture demonstrated outstanding performance across multiple challenging conditions, including low SNR and low sampling rate environments. By leveraging transfer learning, the model achieved faster convergence and superior accuracy even when trained on small datasets, highlighting its practical applicability.

Key findings from this work highlight the effectiveness of the proposed TransConvNet model in addressing challenges associated with wireless signal recognition. The model achieved an accuracy of 92.1%, surpassing baseline approaches such as LSTM, CNN, and RNN, while also exhibiting lower RMSE and variance values. Its robustness to noise was evident as it consistently maintained high accuracy across varying SNR levels, demonstrating adaptability to real-world signal environments. Furthermore, the integration of transfer learning significantly reduced training time and loss, enabling strong performance even with limited labeled data.

Despite these promising results, further research can explore several directions to enhance the model’s applicability. Real-time deployment strategies for edge computing and IoT applications could be developed to enable practical use in real-world scenarios. Reducing the computational complexity of the Transformer module would make the model more efficient for resource-constrained devices. Additionally, expanding the dataset to include more diverse signal types and noise environments could improve its generalization capability. Finally, adaptive transfer learning strategies could be investigated to address performance challenges under domain shifts or new signal classes.

In summary, TransConvNet provides a robust and effective solution for wireless signal recognition, tackling key challenges in noisy, low-sampling-rate environments. With further optimizations, the model holds significant potential to advance wireless communication and signal processing technologies.

## Figures and Tables

**Table 1:** Performance comparison of different models.

Model	Accuracy (%)	RMSE	$R^{2}$	Variance
TransConvNet	92.1	0.84	0.45	0.75
LSTM	88.2	1.01	0.33	0.65
CNN	86.9	1.04	0.31	0.63
RNN	83.5	1.24	0.21	0.48

## Data Availability

Data are contained within the article.
